# 1-[2-(4-Chloro­phen­yl)-5-phenyl-2,3-dihydro-1,3,4-oxadiazol-3-yl]ethanone

**DOI:** 10.1107/S1600536812023100

**Published:** 2012-05-26

**Authors:** Hoong-Kun Fun, Suhana Arshad, P. C. Shyma, Balakrishna Kalluraya, T. Arulmoli

**Affiliations:** a X-ray Crystallography Unit, School of Physics, Universiti Sains Malaysia, 11800 USM, Penang, Malaysia; b Department of Studies in Chemistry, Mangalore University, Mangalagangothri 574 199, Karnataka, India; c SeQuent Scientific Limited, No: 120 A & B, Industrial Area, Baikampady, New Mangalore, Karnataka 575 011, India

## Abstract

In the title compound, C_16_H_14_ClN_3_O_2_, the 2,3-dihydro-1,3,4-oxadiazole ring [maximum deviation = 0.030 (1) Å] and the pyridine ring [maximum deviation = 0.012 (1) Å] are inclined slightly to one another, making a dihedral angle of 11.91 (5)°. The chloro-substituted phenyl ring is almost perpendicular to the 2,3-dihydro-1,3,4-oxadiazole and pyridine rings at dihedral angles of 86.86 (5) and 75.26 (5)°, respectively. In the crystal, π–π [centroid–centroid distance = 3.7311 (6) Å] and C—H⋯π inter­actions are observed.

## Related literature

For the biological activity of 3-acetyl-2,5-disubstituted-2,3-dihydro-1,3,4-oxadiazo­line ring systems, see: Rakesh & Prabhakar (2009 ▶); Priya *et al.* (2007 ▶); Bhatia & Gupta (2011 ▶); Vijesh *et al.* (2011 ▶); Galil & Amr (2000 ▶). For related structures, see: Yehye *et al.* (2010 ▶); Ono *et al.* (2009 ▶). For stability of the temperature controller used in the data collection, see: Cosier & Glazer (1986 ▶).
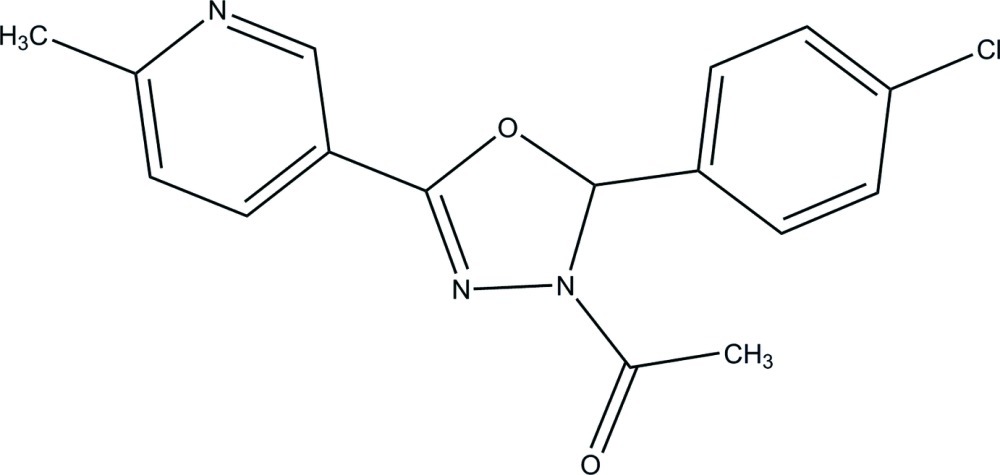


## Experimental

### 

#### Crystal data

C_16_H_14_ClN_3_O_2_
*M* *_r_* = 315.75
Triclinic, 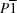

*a* = 5.8623 (2) Å
*b* = 10.9912 (5) Å
*c* = 12.2815 (5) Å
α = 68.214 (1)°
β = 84.707 (1)°
γ = 87.623 (1)°
*V* = 731.67 (5) Å^3^
*Z* = 2
Mo *K*α radiation
μ = 0.27 mm^−1^
*T* = 100 K
0.40 × 0.22 × 0.14 mm

#### Data collection

Bruker SMART APEXII DUO CCD diffractometer
Absorption correction: multi-scan (*SADABS*; Bruker, 2009 ▶) *T*_min_ = 0.899, *T*_max_ = 0.962
19562 measured reflections
5301 independent reflections
4768 reflections with *I* > 2σ(*I*)
*R*_int_ = 0.021

#### Refinement

*R*[*F* ^2^ > 2σ(*F* ^2^)] = 0.037
*wR*(*F* ^2^) = 0.116
*S* = 1.02
5301 reflections
195 parameters
H-atom parameters constrained
Δρ_max_ = 0.60 e Å^−3^
Δρ_min_ = −0.64 e Å^−3^

### 

Data collection: *APEX2* (Bruker, 2009 ▶); cell refinement: *SAINT* (Bruker, 2009 ▶); data reduction: *SAINT*; program(s) used to solve structure: *SHELXTL* (Sheldrick, 2008 ▶); program(s) used to refine structure: *SHELXTL*; molecular graphics: *SHELXTL*; software used to prepare material for publication: *SHELXTL* and *PLATON* (Spek, 2009 ▶).

## Supplementary Material

Crystal structure: contains datablock(s) global, I. DOI: 10.1107/S1600536812023100/hb6803sup1.cif

Structure factors: contains datablock(s) I. DOI: 10.1107/S1600536812023100/hb6803Isup2.hkl

Supplementary material file. DOI: 10.1107/S1600536812023100/hb6803Isup3.cml

Additional supplementary materials:  crystallographic information; 3D view; checkCIF report

## Figures and Tables

**Table 1:** Hydrogen-bond geometry (Å, °) *Cg*3 is the centroid of the C8–C13 benzene ring.

*D*—H⋯*A*	*D*—H	H⋯*A*	*D*⋯*A*	*D*—H⋯*A*
C16—H16*A*⋯*Cg*3^i^	0.98	2.65	3.4360 (13)	138

Symmetry code: (i) 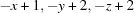.

**Fig. 1.**
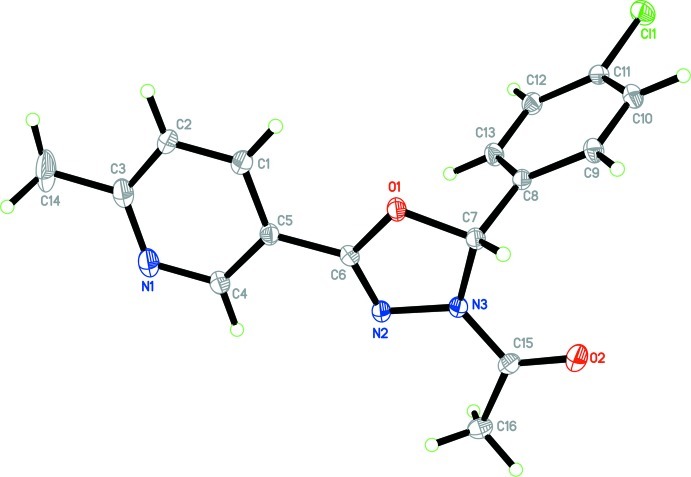
The molecular structure of the title compound, showing 50% probability displacement ellipsoids.

**Fig. 2.**
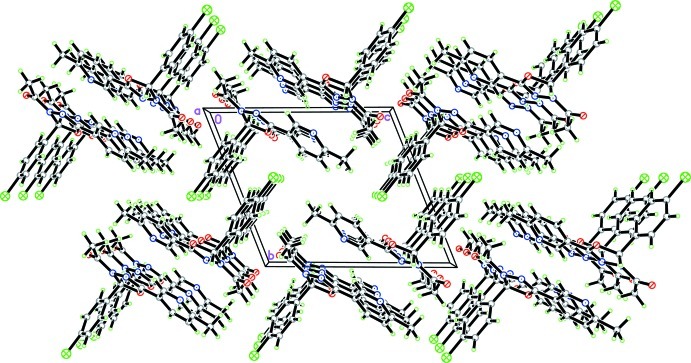
A packing diagram of the title compound viewed along the a axis.

## supplementary crystallographic information

### Comment

Oxadiazole, a five-membered heterocyclic nucleus, has attracted a wide attention of the chemists in search for the new therapeutic molecules. A number of therapeutic agents such as HIV-integrase inhibitor Raltegravir, a nitrofuran antibacterial Furamizole, antihypertensive agents like Tiodazosin and Nesapidil are based on the 1,3,4-oxadiazole moiety. The 3-acetyl-2,5-disubstituted-2,3- dihydro-1,3,4-oxadiazoline ring systems are associated with diverse biological properties such as analgesic, anti-inflammatory, anticancer, anti-HIV, antibacterial, antitubercular activities (Rakesh & Prabhakar, 2009; Priya *et al.*, 2007; Bhatia & Gupta, 2011). Further, substituted pyridines have showed significant biological activities (Vijesh *et al.*, 2011; Galil & Amr, 2000). Pyridine-derived pharmaceuticals include Atazanavir and Imatinib mesylate which are recommended for the treatment of HIV and chronic myelogenous leukemia respectively. Keeping in view of the therapeutic importance of 1,3,4-oxadiazoles and pyridines, we synthesized the title compound to study its crystal structure.

In the molecular structure (Fig. 1), the 2,3-dihydro-1,3,4-oxadiazole ring [O1/N2/N3/C6/C7, with a maximum deviation of 0.030 (1) Å at atom C7] and the pyridine ring [N1/C1–C5, with a maximum deviation of 0.012 (1) Å at atom C3 and C5] are slightly inclined to one another, making a dihedral angle of 11.91 (5)°. Meanwhile, the chloro-substituted phenyl ring (C8–C13) is almost perpendicular to the 2,3-dihydro-1,3,4-oxadiazole and pyridine rings at dihedral angles of 86.86 (5) and 75.26 (5)°, respectively. Bond lengths and angles are within normal ranges and are comparable to related structures (Yehye *et al.*, 2010; Ono *et al.*, 2009).

The crystal packing is shown in Fig. 2. π–π interactions are observed with centroid to centroid distance *Cg*1···*Cg*2 = 3.7311 (6) Å; symmetry code: 1 - *x*, 2 - *y*,1 - *z*. The crystal structure also features intermolecular C16—H16A···*Cg*3 (Table 1) interactions (*Cg*1, *Cg*2 and *Cg*3 are the centroids of O1/N2/N3/C6/C7, N1/C1–C5 and C8–C13 rings, respectively).

### Experimental

Schiff base, *N*'-[(1*E*)-(4-chlorophenyl)methylene]-4- methylbenzohydrazide (0.5 g, 0.0018 mol) was refluxed with acetic anhydride (3 ml) for 1 h. After the completion of reaction, the excess acetic anhydride was distilled out at reduced pressure and the residue obtained was poured into ice cold water. The solid that was separated out was filtered, washed with water and dried. The crude product was recrystallized from hot ethanol in the form of yellow blocks (0.38 g, 76%). *M.p.*: 395–397 K.

### Refinement

All H atoms were positioned geometrically [C–H = 0.95 or 1.00 Å] and refined using a riding model with *U*_iso_(H) = 1.2 or 1.5 *U*_eq_(C). A rotating group model was applied to the methyl groups. The same *U^ij^* parameter was used for atoms pair N1/C3. Three outliers (-2 0 2, -2 0 1 and -2 1 1) were omitted in the final refinement.

### Crystal data

**Table:**

C_16_H_14_ClN_3_O_2_	*Z* = 2
*M**_r_* = 315.75	*F*(000) = 328
Triclinic, *P*1	*D*_x_ = 1.433 Mg m^−^^3^
Hall symbol: -P 1	Mo *K*α radiation, λ = 0.71073 Å
*a* = 5.8623 (2) Å	Cell parameters from 9976 reflections
*b* = 10.9912 (5) Å	θ = 3.1–32.6°
*c* = 12.2815 (5) Å	µ = 0.27 mm^−^^1^
α = 68.214 (1)°	*T* = 100 K
β = 84.707 (1)°	Block, yellow
γ = 87.623 (1)°	0.40 × 0.22 × 0.14 mm
*V* = 731.67 (5) Å^3^	

### Data collection

**Table:**

Bruker SMART APEXII DUO CCD diffractometer	5301 independent reflections
Radiation source: fine-focus sealed tube	4768 reflections with *I* > 2σ(*I*)
Graphite monochromator	*R*_int_ = 0.021
φ and ω scans	θ_max_ = 32.6°, θ_min_ = 2.0°
Absorption correction: multi-scan (*SADABS*; Bruker, 2009)	*h* = −8→8
*T*_min_ = 0.899, *T*_max_ = 0.962	*k* = −16→16
19562 measured reflections	*l* = −18→18

### Refinement

**Table:**

Refinement on *F*^2^	Primary atom site location: structure-invariant direct methods
Least-squares matrix: full	Secondary atom site location: difference Fourier map
*R*[*F*^2^ > 2σ(*F*^2^)] = 0.037	Hydrogen site location: inferred from neighbouring sites
*wR*(*F*^2^) = 0.116	H-atom parameters constrained
*S* = 1.02	*w* = 1/[σ^2^(*F*_o_^2^) + (0.0681*P*)^2^ + 0.3111*P*] where *P* = (*F*_o_^2^ + 2*F*_c_^2^)/3
5301 reflections	(Δ/σ)_max_ = 0.003
195 parameters	Δρ_max_ = 0.60 e Å^−^^3^
0 restraints	Δρ_min_ = −0.64 e Å^−^^3^

### Special details

**Table:**

Experimental. The crystal was placed in the cold stream of an Oxford Cryosystems Cobra open-flow nitrogen cryostat (Cosier & Glazer, 1986) operating at 100.0 (1) K.
Geometry. All e.s.d.'s (except the e.s.d. in the dihedral angle between two l.s. planes) are estimated using the full covariance matrix. The cell e.s.d.'s are taken into account individually in the estimation of e.s.d.'s in distances, angles and torsion angles; correlations between e.s.d.'s in cell parameters are only used when they are defined by crystal symmetry. An approximate (isotropic) treatment of cell e.s.d.'s is used for estimating e.s.d.'s involving l.s. planes.
Refinement. Refinement of *F*^2^ against ALL reflections. The weighted *R*-factor *wR* and goodness of fit *S* are based on *F*^2^, conventional *R*-factors *R* are based on *F*, with *F* set to zero for negative *F*^2^. The threshold expression of *F*^2^ > σ(*F*^2^) is used only for calculating *R*-factors(gt) *etc*. and is not relevant to the choice of reflections for refinement. *R*-factors based on *F*^2^ are statistically about twice as large as those based on *F*, and *R*- factors based on ALL data will be even larger.

### Fractional atomic coordinates and isotropic or equivalent isotropic displacement parameters (Å^2^)

**Table:**

	*x*	*y*	*z*	*U*_iso_*/*U*_eq_	
Cl1	0.93968 (5)	0.44230 (2)	1.24269 (2)	0.02345 (8)	
O1	0.75357 (12)	0.84198 (7)	0.70235 (6)	0.01543 (13)	
O2	0.71195 (14)	1.07742 (8)	0.89561 (7)	0.02114 (16)	
N1	0.14662 (17)	0.82200 (10)	0.47108 (8)	0.02164 (14)	
N2	0.42483 (14)	0.94931 (8)	0.72117 (7)	0.01425 (14)	
N3	0.57912 (14)	0.96760 (8)	0.79406 (7)	0.01478 (15)	
C1	0.56078 (19)	0.72732 (11)	0.56195 (10)	0.0218 (2)	
H1A	0.7044	0.6960	0.5920	0.026*	
C2	0.4694 (2)	0.67544 (11)	0.49036 (10)	0.0221 (2)	
H2A	0.5479	0.6079	0.4709	0.026*	
C3	0.26192 (19)	0.72257 (11)	0.44693 (9)	0.02164 (14)	
C4	0.23766 (16)	0.87227 (10)	0.54253 (9)	0.01640 (17)	
H4A	0.1587	0.9408	0.5601	0.020*	
C5	0.44533 (16)	0.82564 (9)	0.59111 (8)	0.01382 (16)	
C6	0.53469 (15)	0.87669 (9)	0.67240 (8)	0.01324 (15)	
C7	0.79357 (15)	0.89356 (9)	0.79165 (8)	0.01384 (16)	
H7A	0.9281	0.9536	0.7659	0.017*	
C8	0.82979 (15)	0.78264 (9)	0.90601 (8)	0.01289 (15)	
C9	1.02946 (15)	0.77444 (10)	0.96167 (8)	0.01520 (16)	
H9A	1.1425	0.8404	0.9281	0.018*	
C10	1.06480 (16)	0.67008 (10)	1.06622 (9)	0.01629 (17)	
H10A	1.2006	0.6646	1.1045	0.020*	
C11	0.89815 (16)	0.57433 (9)	1.11339 (8)	0.01493 (16)	
C12	0.69506 (17)	0.58158 (10)	1.06022 (9)	0.01712 (17)	
H12A	0.5814	0.5160	1.0944	0.021*	
C13	0.66228 (16)	0.68659 (10)	0.95626 (9)	0.01628 (17)	
H13A	0.5248	0.6931	0.9191	0.020*	
C14	0.1547 (3)	0.66352 (15)	0.37211 (12)	0.0388 (3)	
H14A	0.0095	0.6224	0.4122	0.058*	
H14B	0.2585	0.5973	0.3588	0.058*	
H14C	0.1261	0.7322	0.2964	0.058*	
C15	0.55511 (17)	1.06142 (9)	0.84241 (8)	0.01540 (16)	
C16	0.33496 (18)	1.13830 (10)	0.82619 (10)	0.02050 (19)	
H16A	0.3508	1.2156	0.8465	0.031*	
H16B	0.2109	1.0833	0.8775	0.031*	
H16C	0.2994	1.1662	0.7440	0.031*	

### Atomic displacement parameters (Å^2^)

**Table:**

	*U*^11^	*U*^22^	*U*^33^	*U*^12^	*U*^13^	*U*^23^
Cl1	0.02985 (14)	0.01791 (12)	0.01908 (12)	0.00100 (9)	−0.00754 (9)	−0.00162 (9)
O1	0.0136 (3)	0.0209 (3)	0.0149 (3)	0.0038 (2)	−0.0041 (2)	−0.0101 (3)
O2	0.0226 (3)	0.0219 (4)	0.0229 (4)	−0.0025 (3)	−0.0045 (3)	−0.0119 (3)
N1	0.0243 (3)	0.0241 (3)	0.0164 (3)	−0.0039 (2)	−0.0053 (2)	−0.0062 (2)
N2	0.0145 (3)	0.0157 (3)	0.0139 (3)	0.0009 (3)	−0.0034 (2)	−0.0065 (3)
N3	0.0151 (3)	0.0162 (3)	0.0158 (3)	0.0032 (3)	−0.0046 (3)	−0.0087 (3)
C1	0.0233 (5)	0.0244 (5)	0.0237 (5)	0.0070 (4)	−0.0089 (4)	−0.0151 (4)
C2	0.0275 (5)	0.0232 (5)	0.0224 (5)	0.0061 (4)	−0.0094 (4)	−0.0153 (4)
C3	0.0243 (3)	0.0241 (3)	0.0164 (3)	−0.0039 (2)	−0.0053 (2)	−0.0062 (2)
C4	0.0162 (4)	0.0185 (4)	0.0146 (4)	0.0005 (3)	−0.0031 (3)	−0.0059 (3)
C5	0.0153 (4)	0.0147 (4)	0.0117 (4)	−0.0003 (3)	−0.0019 (3)	−0.0051 (3)
C6	0.0127 (3)	0.0143 (4)	0.0121 (4)	0.0008 (3)	−0.0021 (3)	−0.0040 (3)
C7	0.0133 (3)	0.0160 (4)	0.0136 (4)	0.0010 (3)	−0.0027 (3)	−0.0068 (3)
C8	0.0123 (3)	0.0144 (4)	0.0133 (4)	0.0008 (3)	−0.0023 (3)	−0.0066 (3)
C9	0.0121 (3)	0.0190 (4)	0.0151 (4)	−0.0012 (3)	−0.0017 (3)	−0.0067 (3)
C10	0.0133 (4)	0.0200 (4)	0.0159 (4)	0.0006 (3)	−0.0034 (3)	−0.0065 (3)
C11	0.0174 (4)	0.0140 (4)	0.0141 (4)	0.0022 (3)	−0.0030 (3)	−0.0058 (3)
C12	0.0183 (4)	0.0147 (4)	0.0186 (4)	−0.0027 (3)	−0.0037 (3)	−0.0057 (3)
C13	0.0147 (4)	0.0166 (4)	0.0180 (4)	−0.0020 (3)	−0.0046 (3)	−0.0059 (3)
C14	0.0595 (9)	0.0387 (7)	0.0213 (5)	−0.0216 (7)	−0.0116 (5)	−0.0106 (5)
C15	0.0191 (4)	0.0131 (4)	0.0144 (4)	−0.0012 (3)	0.0001 (3)	−0.0059 (3)
C16	0.0223 (4)	0.0185 (4)	0.0235 (5)	0.0051 (3)	−0.0026 (4)	−0.0114 (4)

### Geometric parameters (Å, º)

**Table:**

Cl1—C11	1.7373 (10)	C7—C8	1.5055 (13)
O1—C6	1.3673 (11)	C7—H7A	1.0000
O1—C7	1.4489 (11)	C8—C9	1.3923 (12)
O2—C15	1.2300 (12)	C8—C13	1.3966 (13)
N1—C4	1.3531 (13)	C9—C10	1.3941 (14)
N1—C3	1.3716 (16)	C9—H9A	0.9500
N2—C6	1.2851 (12)	C10—C11	1.3878 (14)
N2—N3	1.3993 (11)	C10—H10A	0.9500
N3—C15	1.3648 (12)	C11—C12	1.3954 (13)
N3—C7	1.4730 (12)	C12—C13	1.3900 (14)
C1—C2	1.3674 (14)	C12—H12A	0.9500
C1—C5	1.3914 (14)	C13—H13A	0.9500
C1—H1A	0.9500	C14—H14A	0.9800
C2—C3	1.3764 (15)	C14—H14B	0.9800
C2—H2A	0.9500	C14—H14C	0.9800
C3—C14	1.4979 (16)	C15—C16	1.5005 (14)
C4—C5	1.3986 (13)	C16—H16A	0.9800
C4—H4A	0.9500	C16—H16B	0.9800
C5—C6	1.4569 (13)	C16—H16C	0.9800
			
C6—O1—C7	106.85 (7)	C13—C8—C7	119.68 (8)
C4—N1—C3	118.61 (9)	C8—C9—C10	120.49 (9)
C6—N2—N3	104.34 (8)	C8—C9—H9A	119.8
C15—N3—N2	124.21 (8)	C10—C9—H9A	119.8
C15—N3—C7	123.06 (8)	C11—C10—C9	118.84 (9)
N2—N3—C7	111.51 (7)	C11—C10—H10A	120.6
C2—C1—C5	120.58 (10)	C9—C10—H10A	120.6
C2—C1—H1A	119.7	C10—C11—C12	121.68 (9)
C5—C1—H1A	119.7	C10—C11—Cl1	119.71 (7)
C1—C2—C3	118.95 (10)	C12—C11—Cl1	118.60 (7)
C1—C2—H2A	120.5	C13—C12—C11	118.69 (9)
C3—C2—H2A	120.5	C13—C12—H12A	120.7
N1—C3—C2	122.02 (10)	C11—C12—H12A	120.7
N1—C3—C14	118.22 (11)	C12—C13—C8	120.56 (9)
C2—C3—C14	119.75 (12)	C12—C13—H13A	119.7
N1—C4—C5	121.54 (9)	C8—C13—H13A	119.7
N1—C4—H4A	119.2	C3—C14—H14A	109.5
C5—C4—H4A	119.2	C3—C14—H14B	109.5
C1—C5—C4	118.25 (9)	H14A—C14—H14B	109.5
C1—C5—C6	121.10 (9)	C3—C14—H14C	109.5
C4—C5—C6	120.63 (8)	H14A—C14—H14C	109.5
N2—C6—O1	116.52 (8)	H14B—C14—H14C	109.5
N2—C6—C5	126.02 (8)	O2—C15—N3	118.81 (9)
O1—C6—C5	117.44 (8)	O2—C15—C16	124.60 (9)
O1—C7—N3	100.48 (7)	N3—C15—C16	116.59 (9)
O1—C7—C8	109.92 (7)	C15—C16—H16A	109.5
N3—C7—C8	113.89 (8)	C15—C16—H16B	109.5
O1—C7—H7A	110.7	H16A—C16—H16B	109.5
N3—C7—H7A	110.7	C15—C16—H16C	109.5
C8—C7—H7A	110.7	H16A—C16—H16C	109.5
C9—C8—C13	119.71 (9)	H16B—C16—H16C	109.5
C9—C8—C7	120.61 (8)		
			
C6—N2—N3—C15	164.62 (9)	C15—N3—C7—O1	−162.77 (8)
C6—N2—N3—C7	−3.06 (10)	N2—N3—C7—O1	5.08 (10)
C5—C1—C2—C3	0.40 (18)	C15—N3—C7—C8	79.78 (11)
C4—N1—C3—C2	−1.88 (16)	N2—N3—C7—C8	−112.37 (9)
C4—N1—C3—C14	177.33 (10)	O1—C7—C8—C9	123.04 (9)
C1—C2—C3—N1	1.52 (18)	N3—C7—C8—C9	−125.11 (9)
C1—C2—C3—C14	−177.68 (11)	O1—C7—C8—C13	−56.48 (11)
C3—N1—C4—C5	0.33 (15)	N3—C7—C8—C13	55.37 (11)
C2—C1—C5—C4	−1.86 (16)	C13—C8—C9—C10	0.93 (14)
C2—C1—C5—C6	176.61 (10)	C7—C8—C9—C10	−178.58 (8)
N1—C4—C5—C1	1.50 (15)	C8—C9—C10—C11	0.34 (14)
N1—C4—C5—C6	−176.97 (9)	C9—C10—C11—C12	−1.41 (15)
N3—N2—C6—O1	−0.56 (11)	C9—C10—C11—Cl1	179.25 (7)
N3—N2—C6—C5	177.71 (9)	C10—C11—C12—C13	1.16 (15)
C7—O1—C6—N2	3.92 (11)	Cl1—C11—C12—C13	−179.50 (8)
C7—O1—C6—C5	−174.50 (8)	C11—C12—C13—C8	0.16 (15)
C1—C5—C6—N2	−166.88 (10)	C9—C8—C13—C12	−1.19 (15)
C4—C5—C6—N2	11.55 (15)	C7—C8—C13—C12	178.33 (9)
C1—C5—C6—O1	11.37 (14)	N2—N3—C15—O2	−172.90 (9)
C4—C5—C6—O1	−170.20 (8)	C7—N3—C15—O2	−6.60 (14)
C6—O1—C7—N3	−5.07 (9)	N2—N3—C15—C16	7.16 (14)
C6—O1—C7—C8	115.27 (8)	C7—N3—C15—C16	173.46 (9)

### Hydrogen-bond geometry (Å, º)

*Cg*3 is the centroid of the C8–C13 benzene ring.

**Table:**

*D*—H···*A*	*D*—H	H···*A*	*D*···*A*	*D*—H···*A*
C16—H16*A*···*Cg*3^i^	0.98	2.65	3.4360 (13)	138

Symmetry code: (i) −*x*+1, −*y*+2, −*z*+2.
